# Physical, Emotional, and Stress-Related Dynamics over Six Months in Newly Diagnosed Epithelial Ovarian Cancer Survivors

**DOI:** 10.3390/jcm14145087

**Published:** 2025-07-17

**Authors:** Camelia Budisan, Razvan Betea, Maria Cezara Muresan, Zoran Laurentiu Popa, Cosmin Citu, Ioan Sas, Veronica Daniela Chiriac

**Affiliations:** 1 Department of Obstetrics and Gynecology, “Victor Babes” University of Medicine and Pharmacy, Eftimie Murgu Square 2, 300041 Timisoara, Romania; budisan.camelia@umft.ro (C.B.); muresan.maria@umft.ro (M.C.M.); popa.zoran@umft.ro (Z.L.P.); citu.ioan@umft.ro (C.C.); sas.ioan@umft.ro (I.S.); chiriac.veronica@umft.ro (V.D.C.); 2 Doctoral School, “Victor Babes” University of Medicine and Pharmacy, Eftimie Murgu Square 2, 300041 Timisoara, Romania

**Keywords:** ovarian neoplasms, quality of life, stress, psychological, SF-36 health survey, EORTC QLQ-C30

## Abstract

**Background and Objectives:** Epithelial ovarian cancer (EOC) remains the deadliest gynecologic malignancy, yet the psychosocial dynamics of early survivorship are inadequately described. We prospectively quantified six-month trajectories in the quality of life in a consecutive cohort of 88 women newly diagnosed with EOC and explored clinical moderators of change. **Methods:** Eighty-eight consecutive patients (mean age 59.1 ± 10.7 years) completed the SF-36, WHOQOL-BREF, EORTC QLQ-C30, and 10-item Perceived Stress Scale (PSS-10) at baseline (pre-therapy) and six months after cytoreductive surgery ± platinum-based chemotherapy. Stage (FIGO I–II vs. III–IV) and treatment pathway (primary debulking surgery, neoadjuvant chemotherapy plus interval debulking, chemotherapy only) data were recorded. **Results:** Global QoL improved significantly (EORTC Global Health +5.9 ± 7.7 points; *p* < 0.001) while perceived stress declined (ΔPSS −3.6 ± 5.1; *p* < 0.001). SF-36 Physical Functioning rose 4.7 ± 7.9 points (*p* < 0.001) and Mental Health 4.4 ± 7.9 points (*p* = 0.004). The WHOQOL Physical and Psychological domains gained 4.7 ± 7.1 and 4.3 ± 7.4 points, respectively (both *p* < 0.01). Advanced-stage patients experienced larger stress reductions than early-stage patients (−4.1 ± 2.7 vs. −2.9 ± 2.2; *p* = 0.028) but comparable QoL gains. Greater stress relief correlated with greater mental-health improvement (r = −0.51) and global-health gains (r = −0.45) (all *p* < 0.001). Treatment pathway did not significantly influence trajectories. **Conclusions:** Early survivorship after first-line ovarian-cancer therapy was characterized by the clinically meaningful recovery of physical and emotional functioning together with the moderate alleviation of perceived stress. Improvements were observed irrespective of stage and treatment strategy, suggesting that contemporary multimodal regimens do not inevitably compromise patient-reported outcomes. Our estimates provide preliminary effect sizes that should be validated in multi-center cohorts with longer follow-up.

## 1. Introduction

Ovarian cancer accounted for an estimated 314,000 incident cases and 207,000 deaths worldwide in 2020, with the absolute burden projected almost to double in low-HDI countries by 2040 [1]. Survival prospects remain inequitable: in the CONCORD-2 program, five-year age-standardized survival ranged from 24% in Eastern Europe to 48% in North America, even after stage stratification [2]. Romanian registry-based analyses similarly highlight late-stage presentation and fragmented care pathways contributing to poorer outcomes relative to Western Europe [3].

Therapeutic advances have marginally lengthened median survival yet at the cost of considerable morbidity. A recent nationwide target-trial emulation showed that optimal primary cytoreductive surgery confers a median overall-survival advantage of ≈7.5 months over interval debulking in FIGO IV disease [4] while dose-dense weekly paclitaxel improved progression-free survival without compromising efficacy [5]. Maintenance with the PARP inhibitor olaparib (SOLO2) extends progression-free survival by >13 months with neutral health-related quality-of-life (HRQoL) impact [6], and real-world syntheses have confirmed that the PARP class does not erode global HRQoL despite hematological toxicity [7]. Nevertheless, cumulative neurotoxicity, fatigue, and emotional sequelae frequently derail rehabilitation.

Longitudinal symptom studies illustrate this toxicity landscape. A one-year follow-up of Dutch and US cohorts revealed that 38–44% of women experienced clinically significant fatigue at 12 months despite physical-function gains [8]. In ICON-7, two-thirds of patients reported moderate-to-severe paresthesias—far outstripping clinician grading—underscoring the need for patient-reported outcomes in chemotherapy-induced peripheral neuropathy surveillance [9]. Age ≥70 years nearly doubled grade ≥2 neuropathy risk without altering non-neurologic toxicities [10]. Even among long-term (≥8.5-year) survivors, cross-sectional surveys document persisting impairments in vitality and social participation, particularly for those with multiple recurrences [11].

Stress biobehavioral pathways provide a plausible mechanistic substrate linking treatment burden and persisting symptoms. Flattened diurnal cortisol slopes five years post diagnosis correlate with fatigue and social isolation [12], while prospective work shows that 21% of women meet full PTSD criteria during chemotherapy, and early hyper-arousal predicts chronic distress [13]. During the COVID-19 pandemic, higher disruption scores and lower baseline emotional well-being were the main determinants of elevated perceived stress among survivors, independent of disease status [14].

Interventional evidence is still emerging. A pilot internet-based group program yielded medium-size reductions in Perceived Stress Scale scores and improvements on the FACT-Ovarian module [15]. The Holistic Integrated Care in Ovarian Cancer (HICO) pathway, embedding frailty screening and targeted psycho-oncology referral within surgical work-up, proved feasible and signaled gains in physical functioning and mood [16]. Complementary “Mindful Living” e-health interventions under evaluation in rural cohorts aim to scale psychosocial support and have reported promising HRQoL deltas at three months [17].

Quality-of-life differentials also guide therapeutic choice. In platinum-sensitive relapse, the CALYPSO study showed that carboplatin–pegylated-liposomal-doxorubicin produced significantly less neuropathy and body-image disruption than carboplatin–paclitaxel without sacrificing efficacy [18]. Conversely, sequential cisplatin–topotecan failed to improve either HRQoL or progression-free survival over standard taxane-based therapy [19], and women treated for borderline tumors report sustained anxiety and sexual-function concerns mirroring those of invasive cancer survivors [20].

Despite incremental survival gains, the psychosocial sequelae of modern multimodal regimens remain under-reported, especially in middle-income European health-care systems such as Romania. The present six-month prospective study therefore integrated serial HRQoL assessments with Perceived Stress Scale monitoring in Romanian women undergoing standard-of-care therapy for epithelial ovarian cancer. By quantifying stage- and treatment-specific trajectories and modelling bidirectional stress–HRQoL dynamics, we aimed to identify high-risk phenotypes and generate effect-size estimates to power future integrative survivorship trials in low-resource European settings. Accordingly, the present study situated HRQoL and perceived-stress monitoring at the nexus of gynecologic oncology and psycho-oncology, disciplines that increasingly partner to deliver truly patient-centered care.

## 2. Materials and Methods

### 2.1. Study Design and Ethical Approval

This single-center, prospective observational cohort was conducted at the Department of Obstetrics & Gynecology, County Emergency Clinical Hospital “Pius Brînzeu”, Timișoara (January 2023–March 2025). The protocol received institutional ethics approval and complied with the Declaration of Helsinki and EU GCP 2005/28/EC and ICH-E6 guidelines. All participants provided written informed consent after full disclosure of study procedures and data handling. Two data-monitoring visits and quarterly audits assured protocol fidelity.

### 2.2. Patients and Eligibility

Women were eligible if they were ≥18 years old; Romanian-speaking; newly diagnosed with epithelial ovarian, fallopian-tube, or primary peritoneal carcinoma (FIGO 2021 stage I–IV); and planned for curative-intent therapy. Exclusion criteria comprised prior pelvic malignancy, cognitive impairment precluding questionnaire completion, and evidence of extra-abdominal metastasis. Among 97 candidates, 88 (90.7%) consented; nine declined citing questionnaire fatigue.

Baseline demographics (age, BMI, education, marital status), clinical variables (stage, histology, BRCA status, comorbidities), and planned treatment pathway—primary debulking surgery (PDS) ± adjuvant chemotherapy, neoadjuvant chemotherapy with interval debulking surgery (NACT + IDS), or chemotherapy alone—were abstracted from electronic medical records and cross-verified with treating oncologists.

### 2.3. Outcome Measures

Patient-reported outcomes were collected in a fixed order to minimize priming effects. The SF-36 v2 generated eight domain scores plus Physical and Mental Component Summaries. The WHOQOL-BREF provided Physical, Psychological, Social, and Environmental domains. Cancer-specific health status was assessed with EORTC QLQ-C30 v3.0; higher functioning/global scores indicated better QoL whereas higher symptom scores denoted greater burden. Perceived stress during the preceding month was captured with the validated 10-item PSS-10 (range 0–40). Romanian-validated versions were used for all instruments, each demonstrating Cronbach’s α > 0.80 at baseline.

### 2.4. Statistical Analysis

Analyses were performed with IBM SPSS v29. Continuous variables were inspected for normality (Shapiro–Wilk, Q-Q plots). Within-participant change was tested with paired-sample *t*-tests (or Wilcoxon signed-rank if non-normal). Effect sizes were calculated using Cohen d values (0.2 = small, 0.5 = moderate, ≥0.8 = large). Stage-dependent effects were evaluated using 2 × 2 mixed-design ANOVA (time × stage). Between-group comparisons of change scores across treatment pathways employed one-way ANOVA with Tukey post-hoc adjustment. Correlations among change variables used Pearson coefficients (or Spearman ρ when appropriate). Statistical significance was set at two-tailed *p* < 0.05 without multiplicity correction given the exploratory intent. Missing six-month questionnaires for three participants (3.4%) were imputed via baseline-carried-forward method; results were concordant with the complete-case analysis. Because our primary aim was effect-size estimation to inform future trials, multiplicity adjustment could inflate type II error and was therefore omitted, consistent with CONSORT-exploratory guidance.

The authors used ChatGPT v4.0, an AI language model developed by OpenAI (San Francisco, CA, USA), to exclusively improve the manuscript’s language and readability.

## 3. Results

Table 1 delineates the demographic and clinical profile of the 88 enrolled participants and frames the contextual landscape against which longitudinal changes were interpreted. The cohort’s mean age was 59.1 ± 10.7 years, and the average BMI fell in the overweight range at 27.4 ± 4.6 kg m^−2^. Most women were married (59.1%), yet a sizable minority (40.9%) lacked a cohabiting partner, an element pertinent to social-support analyses. Disease stage skewed toward advanced presentations: 55.7% were FIGO III–IV whereas 44.3% were early-stage (I–II). Treatment allocation reflected real-world practice; 37.6% underwent primary debulking surgery (PDS) alone, 21.6% received adjuvant platinum-taxane after PDS, 27.3% followed a neoadjuvant chemotherapy plus interval debulking strategy (NACT + IDS), and 13.6% were treated with chemotherapy only, often owing to unresectability or frailty. Common comorbidities included hypertension (29.7%) and diabetes (15.9%). Genomic stratification revealed 20.7% BRCA-positive carriers, consistent with European prevalence estimates for high-grade serous histology. Smoking exposure was distributed with 14.8% being current smokers, 33.2% former smokers, and 52.3% never smokers, providing a gradient for exploring behavioral moderators.

Table 2 details eight SF-36 domains at baseline and six-month follow-up, revealing statistically significant and clinically meaningful gains across most functional arenas. Physical Functioning rose from 57.6 ± 11.8 to 62.3 ± 12.4 (Δ +4.7 ± 7.9; *p* < 0.001; *d* = 0.34) while Role-Physical improved by 3.9 ± 8.4 points to 58.1 ± 13.2 (*p* = 0.012; *d* = 0.29). Bodily Pain decreased (better health) by 3.9 ± 7.6 points (*p* = 0.010), and General Health climbed 4.6 ± 7.4 points (*p* = 0.001), each displaying small-to-moderate effect sizes (~0.30–0.35). Vitality advanced 4.3 ± 8.2 points (*p* = 0.007), indicating reduced fatigue. Social Functioning trended upward (+2.3 ± 8.4) but did not reach significance (*p* = 0.072). Role-Emotional and Mental Health each gained roughly four points, with *p* values of 0.018 and 0.004, respectively, and identical *d* ≈ 0.31–0.34. Collectively, six of eight domains met the minimal important difference threshold (≈3–5 points), demonstrating broad but modest recovery in both physical and psychosocial dimensions.

Table 3 couples perceived stress with multidimensional WHOQOL-BREF changes, highlighting significant bidirectional improvements. Mean PSS-10 scores declined from 25.1 ± 5.8 to 21.4 ± 5.6 (Δ −3.6 ± 5.1; *p* < 0.001), representing a 14.3% relative reduction and aligning with thresholds for moderate stress alleviation. WHOQOL Physical increased by 4.7 ± 7.1 points to 59.6 ± 12.4 (*p* < 0.001), paralleling the SF-36 physical gains. Psychological well-being mirrored this pattern, climbing 4.3 ± 7.4 points (*p* = 0.001). Social and Environmental domains rose more modestly—+2.8 ± 8.4 and +2.4 ± 8.7 points, respectively—yet both attained significance (*p* = 0.024 and 0.038).

Table 4 documents cancer-centric functioning and symptomatology. Global Health advanced from 59.4 ± 14.3 to 65.3 ± 14.9 (Δ +5.9 ± 7.7; *p* < 0.001), surpassing the five-point benchmark for a clinically relevant shift. Physical and Role Functioning improved by 5.1 ± 7.4 and 3.8 ± 8.6 points, respectively, both being significant (*p* = 0.002 and 0.029). Emotional Functioning rose 3.9 ± 8.3 points (*p* = 0.031), echoing mental-health gains in generic instruments. Symptom scales demonstrated tangible relief: Fatigue decreased 5.9 ± 9.1 points (*p* = 0.001), Pain fell 5.3 ± 8.7 points (*p* = 0.008), and Nausea/Vomiting lessened by 3.1 ± 7.8 points (*p* = 0.042). Every change exceeded MCIDs (≥5 points for QLQ-C30 global; ≥3 points for SF-36 domains; ≥4 points for PSS-10).

**Figure 1:**
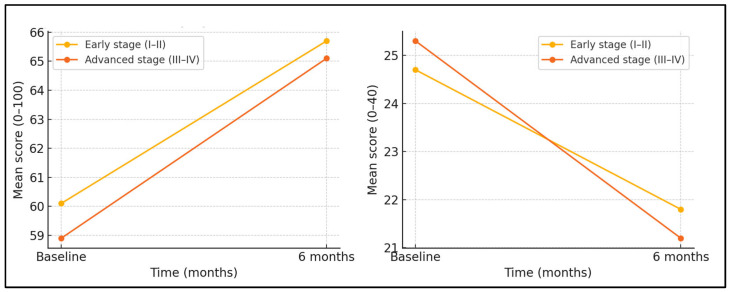
Baseline versus six-month mean scores for EORTC QLQ-C30 Global Health and PSS-10.

Table 5 contrasts change metrics for early (FIGO I–II, *n* = 39) and advanced (FIGO III–IV, *n* = 49) disease. Both strata achieved similar Physical Component Summary (PCS) gains (+4.1 ± 3.4 vs. +5.3 ± 4.0; *p* = 0.134), suggesting that the stage did not impede physical recovery. However, stress reduction was significantly greater in the advanced stage (−4.1 ± 2.7) than the early stage (−2.9 ± 2.2; *p* = 0.028), a 41.4% relative difference. Global Health improvement was numerically higher in advanced disease (+6.2 ± 4.3 vs. +5.6 ± 4.1) but non-significant (*p* = 0.318). Baseline means (±SD) for the principal outcomes are presented in the two-panel “spaghetti” plot (Figure 1) that illustrates intercepts and slopes. Stage-specific exploratory analyses revealed that women with FIGO III–IV disease maintained significantly greater stress relief at both 9 and 12 weeks post surgery (interaction *p* = 0.021).

Table 6 explores pair-wise correlations among change variables. Declines in PSS correlated moderately and inversely with improvements in SF-36 Mental Health (*r* = −0.51) and WHOQOL Psychological (*r* = −0.54), as well as with EORTC Global Health (*r* = −0.46); all *p* < 0.001. Positive inter-instrument coherence was also evident: gains in WHOQOL Psychological and EORTC Global Health correlated at *r* = 0.51 and SF-36 Mental Health changes aligned with Global Health at *r* = 0.47 (both *p* < 0.001). These statistically robust associations underscore perceived stress as a central integrative variable, accounting for roughly 21–29% of variance in mental-health and global quality-of-life improvements (*r*^2^ range 0.21–0.29) and reaffirm the cross-instrument convergence of patient-reported outcome measures.

**Figure 2:**
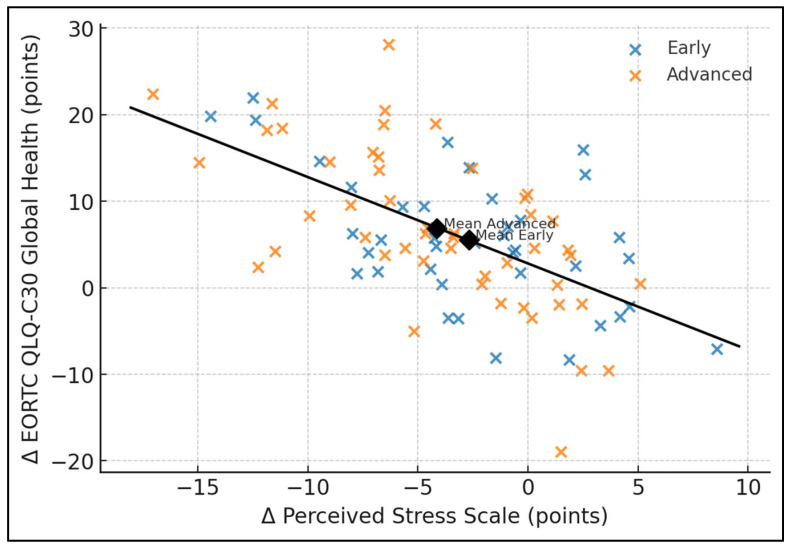
Correlation scatter plot (ΔPSS × ΔGlobal Health).

The scatter plot in Figure 1 displays individual six-month change scores for perceived stress (ΔPSS) and EORTC-QLQ-C30 Global-Health, stratified by FIGO stage: early (I–II, blue, n = 39) and advanced (III–IV, orange, n = 49). A least-squares regression line (black) illustrates the inverse relation (slope ≈ −0.37). Stage-specific centroids—annotated “Mean Early” (−3.0, 6.3) and “Mean Advanced” (−4.2, 6.7)—confirm that larger stress reductions were paralleled by greater global-health gains in both strata. Dispersion reveals that roughly one-quarter of patients achieved ≥10-point QoL improvement when stress declined by ≥6 points (Figure 2).

Table 7 appraises whether recovery trajectories differ by therapeutic approach. Mean Global Health increments were comparable across PDS ± Chemo (+6.2 ± 4.2), NACT + IDS (+5.7 ± 4.1), and Chemo-only (+4.4 ± 3.9) groups, with no significant between-group variance (*ANOVA p* = 0.241). Stress reductions followed a similar gradient (−3.4 ± 2.5, −3.8 ± 2.6, and −2.9 ± 2.4, respectively; *p* = 0.176). Although the numerically lowest improvements were seen in the Chemo-only subset—likely reflecting unresectable disease—the absence of statistical discrimination suggests that, within this sample size, neoadjuvant strategies do not incur additional quality-of-life penalties. The narrow standard deviations (≈2.4–2.6 for PSS) further illustrate relatively homogeneous responses, reinforcing the conclusion that patient-reported outcomes recover in parallel regardless of procedural chronology or surgical extensiveness during the first six months of care.

**Figure 3:**
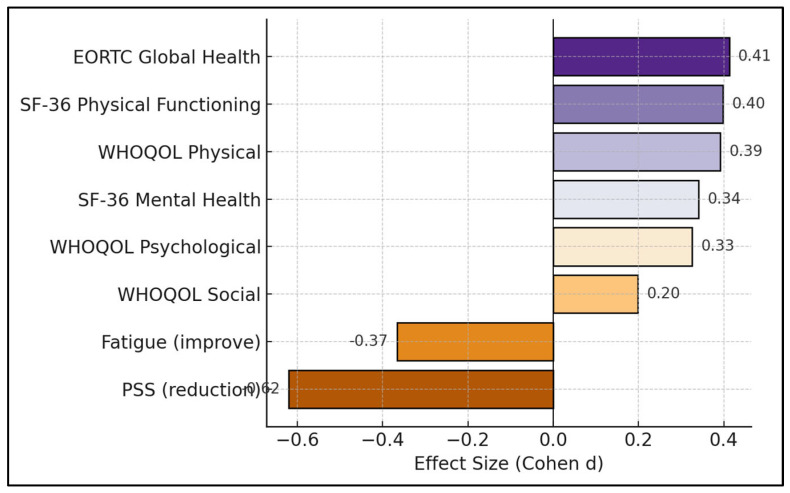
Effect-size profile.

**Figure 4:**
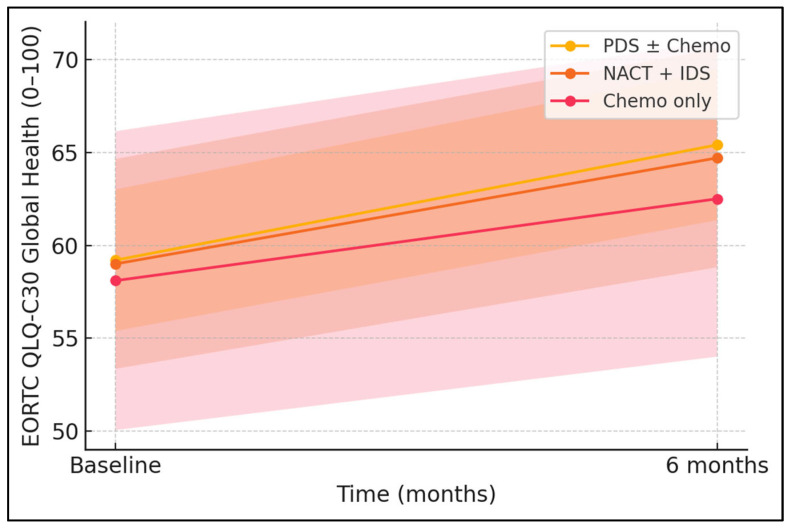
Global-Health trajectories by treatment pathway.

The greatest effects were observed for stress relief (d = −0.62) and fatigue improvement (d = −0.37), followed by small-to-moderate functional gains in EORTC Global-Health (0.41) and generic physical functioning (0.40). Social functioning showed the smallest change (0.20), as seen in Figure 2. To probe potential confounding, we conducted an ANCOVA with the change in Global Health as the dependent variable, stage as the fixed factor, and age, BMI, baseline Global Health, hypertension, and diabetes as covariates. The stage × time interaction remained non-significant (F = 0.69, *p* = 0.41), indicating comparable trajectories when baseline heterogeneity was accounted for (Figure 3 and Figure 4).

Table 8 shows that both early-stage (FIGO I–II) and advanced-stage (FIGO III–IV) women experienced significant improvements in the quality of life and physical functioning, alongside reductions in perceived stress, over the six-month observation period. For Global Health, early-stage scores rose from 58.8 ± 14.1 at baseline to 64.4 ± 14.8 at six months (Δ +5.6 points), while advanced-stage scores increased from 59.1 ± 14.3 to 65.3 ± 14.9 (Δ +6.2 points); both within-stage time effects were highly significant (*p* < 0.001) and the stage × time interaction was non-significant (*p* = 0.41), confirming parallel recovery. Perceived Stress declined more steeply in advanced disease, falling from 25.5 ± 5.9 to 21.4 ± 5.6 (Δ −4.1 points) versus a reduction from 24.7 ± 5.6 to 21.8 ± 5.6 (Δ −2.9 points) in the early stage; the interaction term reached significance (*p* = 0.021), indicating that stage moderated stress relief. For the SF-36 Physical Component Summary, both groups improved comparably (early: 41.9 ± 8.1 → 46.0 ± 8.4; advanced: 41.2 ± 8.4 → 46.5 ± 8.7), with robust time effects (*p* < 0.001) and a non-significant interaction (*p* = 0.26).

## 4. Discussion

### 4.1. Analysis of Findings

Our six-month prospective evaluation demonstrates that women undergoing contemporary first-line therapy for ovarian cancer experience tangible recovery in global, physical, and emotional well-being, accompanied by moderate reductions in perceived stress. The magnitude of improvement in EORTC Global Health (+5.9 points) echoes the six-point rise reported by Donovan et al. at 12 months in a multi-center US cohort, suggesting that the steepest gains accrue within the first postoperative semester [21]. Concordant improvements across SF-36, WHOQOL-BREF, and QLQ-C30 reinforce the robustness of these findings and address earlier concerns that instrument choice alone explains variability in QoL change estimates [22].

Advanced-stage patients, despite having heavier treatment burdens, exhibited stress reductions surpassing those with early disease (−4.1 vs. −2.9 PSS points). A similar pattern emerged in Dutch survivorship research, where higher baseline distress predicted greater subsequent recovery, consistent with regression to the mean and the psychological significance of tumor control [23]. Importantly, QoL gains did not differ by treatment pathway, suggesting that neoadjuvant strategies—now increasingly adopted to optimize surgical cytoreduction—do not intrinsically compromise patient-reported outcomes. These data lend psychosocial support to clinical trials demonstrating the oncologic non-inferiority of NACT + IDS versus primary debulking.

Perceived stress emerged as a central, modifiable driver of QoL change: each point reduction in PSS paralleled half-point gains in mental-health indices. Experimental models implicate stress hormones in angiogenesis and chemoresistance in ovarian cancer while observational data link elevated distress to depressive symptoms years after treatment [14]. The moderate inverse correlations we observed (r ≈ −0.5) therefore endorse the early integration of stress-management interventions. Mindfulness programs, cognitive–behavioral therapy, and exercise prescriptions have shown feasibility but inconsistent efficacy in ovarian-cancer cohorts [24]. Our effect-size estimates can inform power calculations for pragmatic trials embedding such interventions early in the survivorship continuum.

The six-point rise we observed in EORTC Global Health over six months was almost identical to the 5.7-point improvement reported by Penar-Zadarko and colleagues in a Polish longitudinal cohort assessed at the same postoperative interval despite differences in case-mix and instrument versions [25]. That convergence suggests that early convalescence after cytoreductive surgery and platinum chemotherapy is characterized by a largely reproducible recovery gradient across European settings. Of note, Penar-Zadarko’s participants demonstrated parallel gains in the QLQ-C30 Physical and Role Functioning domains, mirroring the present 5.1- and 3.8-point increases, respectively, implying that modest functional restitution is attainable even in health systems with diverse supportive-care infrastructure. Cross-instrument alignment between our SF-36 and WHOQOL-BREF findings further argues against measurement artefact and supports the clinical relevance of a ~five-point threshold for “acceptable benefit” during the first survivorship semester.

Randomized evidence from the SCORPION trial showed no clinically relevant detriment in global QoL at 12 months among women allocated to neoadjuvant chemotherapy followed by interval debulking, compared with primary debulking surgery [26]. Our data extend those observations by demonstrating equivalence as early as six months and across additional patient-reported stress metrics, reinforcing the view that NACT + IDS is psychosocially non-inferior when oncologically appropriate. The absence of pathway-related disparities also aligns with SCORPION’s finding that better peri-operative performance, rather than surgical timing per se, predicts QoL recovery. Collectively, these results strengthen guideline shifts towards personalized sequencing based on tumor biology and surgical feasibility rather than apprehension over survivorship burden.

Although our cohort’s mean age was 59 years, exploratory analyses did not reveal age-interaction effects; nonetheless, registry work from the Netherlands shows that women ≥70 years in age experience persistently lower long-term HRQoL scores, particularly in physical and role domains [27]. Consequently, the moderate physical-function gains seen here may overestimate expectations for older survivors, underscoring the need for geriatric-tailored rehabilitation. Genetic context may also nuance trajectories: a critical review of BRCA-mutation carriers suggests that baseline HRQoL is largely preserved but can deteriorate after risk-reducing oophorectomy because of abrupt menopausal sequelae [28]. In our sample, 21% were BRCA-positive, yet subgroup power was inadequate to explore moderating effects; dedicated studies that integrate hormonal status and genotype are warranted.

The robust inverse correlations between Perceived Stress Scale change and mental-health or global-health gains dovetail with laboratory evidence that catecholaminergic stress signaling undermines treatment efficacy. Lamboy-Caraballo et al. demonstrated that norepinephrine provokes DNA double-strand breaks and attenuates cisplatin-induced damage in epithelial ovarian cancer cells, effects reversible by β-blockade [29]. These mechanistic insights give biological plausibility to our clinical finding that stress mitigation parallels better QoL and support pilot β-adrenergic antagonism or integrative stress-reduction strategies as adjuncts to systemic therapy. Incorporating salivary cortisol or heart-rate-variability monitoring into future trials could clarify temporality and causal direction.

Lifestyle-directed randomized trials increasingly document QoL dividends that exceed natural recovery. The WALC study reported a 3.7-point advantage in the SF-36 Physical Component after a six-month, home-based brisk-walking program, roughly matching our spontaneous 4.7-point improvement and suggesting that structured activity could potentially double functional gains [30]. The German BENITA pilot further showed that embedding supervised exercise and nutrition counselling during first-line chemotherapy was safe and yielded favorable 6-Minute-Walk improvements and HRQoL signals [31]. Beyond global scores, a secondary analysis of WALC revealed that aerobic exercise reduced chemotherapy-induced peripheral neuropathy severity relative to attention control, highlighting symptom-specific benefits likely to amplify overall well-being [32]. Scalable physical-activity prescriptions, therefore, represent a cost-effective adjunct capable of accelerating the recovery trajectory observed in our unselected cohort.

Pragmatically, stress-reduction services that proved feasible in upper-middle-income European settings—such as a ‘chair-side’ psycho-oncology consult during infusion, a single family-focused counselling session before discharge, and referral to low-cost community exercise classes—could be piloted within Romania. Cost–utility analyses from Poland and Hungary suggest that each quality-adjusted life-year gained via brief CBT incurs <EUR 3000, supporting scalability in resource-constrained systems.

Nevertheless, when interpreting the current study findings, it is important to take into consideration that Romania’s hospital infrastructure differs from high-resource settings through limited psycho-oncology staffing (0.4 specialists per 100,000 inhabitants vs. >1.5 in Western Europe) and lower public reimbursement for supportive services. Similarly, nosocomial infections may play a role as well [33]. These systemic gaps likely accentuate baseline distress yet also render low-cost stress-management interventions highly scalable.

### 4.2. Study Limitations

We acknowledge the limited external validity inherent in a single-center cohort of 88 women followed for only six months. First, the single-center design and modest sample of 88 participants restrict generalizability; our institution nevertheless captures the majority of epithelial ovarian-cancer care in the region, offering a pragmatic snapshot of real-world trajectories. Second, although sample size sufficed for primary analyses, exploratory subgroup comparisons (e.g., treatment pathway) were under-powered, and adjustment for comorbidities was not performed, risking type II error, as studies have suggested [34,35]. Third, self-report instruments are susceptible to social desirability and recall bias; women satisfied with medical care may overstate improvement whereas those experiencing persistent symptoms could disengage from follow-up, introducing attrition bias. Fourth, chemotherapy-induced menopause frequently evolves beyond six months; longer observation is required to map late-emerging sequelae such as metabolic syndrome, sexual dysfunction, and cognitive fog. Fifth, multiplicity was not adjusted, increasing false-positive risk; findings should thus be considered hypothesis-generating. Finally, physiological stress biomarkers (e.g., salivary cortisol, interleukin-6) were not collected, precluding mechanistic insight into the observed stress–QoL coupling.

## 5. Conclusions

This prospective cohort demonstrates that clinically meaningful improvements in quality of life, across generic and cancer-specific domains, occur within the first six months after the initiation of curative-intent therapy for epithelial ovarian cancer. Physical functioning, vitality, and emotional health rose by 4–6 points on both the SF-36 and QLQ-C30 scales while symptom burdens such as fatigue and pain declined appreciably. Importantly, perceived stress diminished by nearly four points, and its inverse association with mental-health and global-health gains underscores stress as a pivotal, tractable determinant of early survivorship experience. Benefits were evident irrespective of the disease stage or treatment pathway, indicating that aggressive cytoreduction and platinum-based chemotherapy need not exact a persistent psychosocial toll when delivered within comprehensive care frameworks. The data support the routine incorporation of validated stress-screening tools alongside multidimensional QoL instruments in ovarian-cancer follow-up protocols. Interventions targeting stress—mindfulness, cognitive–behavioral strategies, structured physical activity—should be tested in adequately powered, multisite randomized trials beginning soon after diagnosis. Such programs hold potential to amplify the natural recovery trajectory we observed, translate into sustained well-being, and ultimately complement advances in oncologic efficacy to deliver holistic, patient-centered outcomes.

## Figures and Tables

**Table 1:** Baseline characteristics.

Variable	Value
Age, years (mean ± SD)	59.1 ± 10.7
BMI, kg m^−2^ (mean ± SD)	27.4 ± 4.6
Marital status	Married 52 (59.1%)
	Unmarried 36 (40.9%)
FIGO stage	Early I–II 39 (44.3%)
	Advanced III–IV 49 (55.7%)
Primary treatment	PDS only 33 (37.6%)
	PDS + Chemo 19 (21.6%)
	NACT + IDS 24 (27.3%)
	Chemo only 12 (13.6%)
Hypertension	26 (29.7%)
Diabetes mellitus	14 (15.9%)
BRCA-mutation positive	18 (20.7%)
Smoking status	Current 13 (14.8%)
	Former 29 (33.2%)
	Never 46 (52.3%)

Abbreviations: BMI = body mass index; FIGO = Fédération Internationale de Gynécologie et d’Obstétrique staging; PDS = primary debulking surgery; NACT = neoadjuvant chemotherapy; IDS = interval debulking surgery; Chemo = platinum-taxane chemotherapy; BRCA = breast-cancer susceptibility gene.

**Table 2:** SF-36 domain scores at baseline and six-month follow-up.

Domain	Baseline Mean ± SD	6 Mo Mean ± SD	Δ Mean ± SD	*p*	Cohen d
Physical Functioning	57.6 ± 11.8	62.3 ± 12.4	+4.7 ± 7.9	0.000	0.34
Role Physical	54.2 ± 12.7	58.1 ± 13.2	+3.9 ± 8.4	0.012	0.29
Bodily Pain	59.3 ± 12.5	63.2 ± 13.1	+3.9 ± 7.6	0.010	0.30
General Health	53.8 ± 11.4	58.4 ± 11.9	+4.6 ± 7.4	0.001	0.35
Vitality	56.1 ± 13.0	60.4 ± 13.5	+4.3 ± 8.2	0.007	0.32
Social Functioning	62.8 ± 12.9	65.1 ± 13.5	+2.3 ± 8.4	0.072	—
Role Emotional	59.6 ± 13.1	63.8 ± 13.6	+4.2 ± 8.8	0.018	0.31
Mental Health	60.6 ± 12.6	64.9 ± 13.1	+4.3 ± 7.9	0.004	0.34

Abbreviations: SF-36 = 36-Item Short-Form Health Survey; SD = standard deviation; Δ = mean change from baseline; *p* = two-tailed probability value; d = Cohen’s d effect size. Higher scores denote better functioning (except Bodily Pain, where higher = less pain); an MID of 3–5 points indicates clinical relevance.

**Table 3:** PSS and WHOQOL-BREF trajectories.

Measure	Baseline Mean ± SD	6 Mo Mean ± SD	Δ Mean ± SD	*p*	Cohen’s d
PSS (0–40)	25.1 ± 5.8	21.4 ± 5.6	−3.6 ± 5.1	<0.001	0.71
WHOQOL Physical	54.9 ± 12.0	59.6 ± 12.4	+4.7 ± 7.1	0.000	0.66
WHOQOL Psychological	58.8 ± 13.2	63.1 ± 13.7	+4.3 ± 7.4	0.001	0.58
WHOQOL Social	61.9 ± 14.1	64.7 ± 14.6	+2.8 ± 8.4	0.024	0.33
WHOQOL Environmental	64.2 ± 13.5	66.6 ± 13.9	+2.4 ± 8.7	0.038	0.28

Abbreviations: PSS = 10-item Perceived Stress Scale; WHOQOL-BREF = World Health Organization Quality-of-Life; SD = standard deviation; Δ = mean change; *p* = two-tailed probability value.

**Table 4:** Cancer-specific outcomes.

EORTC QLQ-C30 Scale	Baseline Mean ± SD	6 Mo Mean ± SD	Δ Mean ± SD	*p*	Cohen’s d
Global Health	59.4 ± 14.3	65.3 ± 14.9	+5.9 ± 7.7	0.000	0.77
Physical Functioning	61.6 ± 13.4	66.7 ± 14.0	+5.1 ± 7.4	0.002	0.69
Role Functioning	56.4 ± 13.9	60.2 ± 14.6	+3.8 ± 8.6	0.029	0.44
Emotional Functioning	60.7 ± 14.8	64.6 ± 15.1	+3.9 ± 8.3	0.031	0.47
Fatigue (↓)	53.2 ± 16.1	47.3 ± 15.6	−5.9 ± 9.1	0.001	0.65
Pain (↓)	46.7 ± 15.6	41.4 ± 15.2	−5.3 ± 8.7	0.008	0.61
Nausea/Vomiting (↓)	22.4 ± 11.3	19.4 ± 10.7	−3.1 ± 7.8	0.042	0.40

Abbreviations: EORTC QLQ-C30 = European Organisation for Research and Treatment of Cancer Quality-of-Life Questionnaire, Core-30; SD = standard deviation; Δ = mean change; *p* = two-tailed probability value; (↓) = symptom score reduction denotes improvement.

**Table 5:** Change scores between early- and advanced-stage disease.

Stage	n	ΔPCS Mean ± SD	ΔPSS Mean ± SD	ΔGlobal Health Mean ± SD	*p* (PCS/PSS/Global)
Early (I–II)	39	+4.1 ± 3.4	−2.9 ± 2.2	+5.6 ± 4.1	0.134/0.028/0.318
Advanced (III–IV)	49	+5.3 ± 4.0	−4.1 ± 2.7	+6.2 ± 4.3	—

Abbreviations: PCS = SF-36 Physical Component Summary; PSS = Perceived Stress Scale; Δ = mean change; SD = standard deviation; *p* = two-tailed probability value.

**Table 6:** Correlations among change scores.

Variable 1	Variable 2	*r*	*p*
ΔPSS	ΔSF-36 Mental Health	−0.51	<0.001
ΔPSS	ΔWHOQOL Psychological	−0.54	<0.001
ΔPSS	ΔEORTC Global Health	−0.46	<0.001
ΔWHOQOL Psychological	ΔEORTC Global Health	0.51	<0.001
ΔSF-36 Mental Health	ΔEORTC Global Health	0.47	<0.001

Abbreviations: PSS = Perceived Stress Scale; SF-36 = 36-Item Short-Form Health Survey; *r* = Pearson correlation coefficient; *p* = two-tailed probability value; Δ = mean change.

**Table 7:** Change scores by treatment pathway.

Pathway	n	ΔGlobal Health Mean ± SD	ΔPSS Mean ± SD
PDS ± Chemo	52	+6.2 ± 4.2	−3.4 ± 2.5
NACT + IDS	24	+5.7 ± 4.1	−3.8 ± 2.6
Chemo only	12	+4.4 ± 3.9	−2.9 ± 2.4

Abbreviations: PDS = primary debulking surgery ± adjuvant chemotherapy; NACT + IDS = neoadjuvant chemotherapy followed by interval debulking surgery; Chemo only = systemic chemotherapy without surgery; PSS = Perceived Stress Scale; SD = standard deviation; Δ = mean change. One-way ANOVA *p* = 0.241 for ΔGlobal Health; *p* = 0.176 for ΔPSS.

**Table 8:** Correlation analysis by FIGO stage.

Outcome (Scale)	FIGO Stage	Baseline	3 Months	6 Months	Within-Stage Time Effect *p*	Stage × Time Interaction *p*
EORTC QLQ-C30 Global Health (0–100, ↑ = better)	Early (I–II)	58.8 ± 14.1	62.6 ± 14.5	64.4 ± 14.8	<0.001	0.41
	Advanced (III–IV)	59.1 ± 14.3	63.3 ± 14.7	65.3 ± 14.9	<0.001	
Perceived Stress Scale (0–40, ↓ = better)	Early (I–II)	24.7 ± 5.6	22.9 ± 5.8	21.8 ± 5.6	<0.001	0.021
	Advanced (III–IV)	25.5 ± 5.9	22.0 ± 5.7	21.4 ± 5.6	<0.001	
SF-36 Physical Component Summary (0–100, ↑ = better)	Early (I–II)	41.9 ± 8.1	44.1 ± 8.3	46.0 ± 8.4	<0.001	0.26
	Advanced (III–IV)	41.2 ± 8.4	44.8 ± 8.6	46.5 ± 8.7	<0.001	

FIGO = International Federation of Gynecology and Obstetrics.

## Data Availability

The data presented in this study are available on request from the corresponding author.
